# Insulin-Like Growth Factor 1 Has the Potential to Be Used as a Diagnostic Tool and Treatment Target for Autism Spectrum Disorders

**DOI:** 10.7759/cureus.65393

**Published:** 2024-07-25

**Authors:** Jiamin Shen, Lijuan Liu, Yifan Yang, Miao Zhou, Shan Xu, Wanqing Zhang, Chuanjie Zhang

**Affiliations:** 1 Department of Children Health Care, Jingmen Maternity and Child Health Care Hospital, Jingmen, CHN; 2 Department of Medical Microbiology, School of Basic Medical Sciences, Wuhan University, Wuhan, CHN; 3 Department of Children Health Care, Wuhan Children’s Hospital (Wuhan Maternal and Child Healthcare Hospital) Tongji Medical College, Huazhong University of Science and Technology, Wuhan, CHN

**Keywords:** treatment, mecasermin, trofinetide, diagnostic marker, insulin-like growth factor 1, autism spectrum disorder

## Abstract

Autism spectrum disorder (ASD), a heterogeneous group of neurodevelopmental disorders, is characterized by social impairment and repetitive and stereotypic behaviors. Because of the lack of approved laboratory diagnostic markers and effective therapeutic medications, it is one of the most challenging diseases. Therefore, it is urgent to explore potential diagnosis markers or therapeutic targets. Insulin-like growth factor 1 (IGF-1) is a neurotrophic growth factor that enhances brain development. IGF-1 levels in body fluids are lower in preschool children with ASD than in typically developing children, which may serve as a potential diagnostic marker. In various ASD models associated with genetic or environmental exposure, IGF-1 treatment can improve core symptoms or pathological changes, including neuronal development, neural cell survival, balance of synaptic excitation and inhibition, neuroimmunology, and oxidative stress status. In March 2023 an IGF-1 derivative was approved as the first drug for treating Rett syndrome, an ASD-related neurodevelopmental disorder, to improve fundamental symptoms such as social communication. Thus, in this review, we present accumulating evidence of altered IGF-1 levels in ASD patients and the possible mechanisms, as well as evidence that IGF-1 treatment improves the pathophysiology in various ASD models. IGF-1 has the potential to be an early diagnosis marker and an effective therapeutic for ASD.

## Introduction and background

Autism spectrum disorder (ASD) is a neurodevelopmental disorder that manifests in early childhood and persists throughout the lifespan. Its clinical characteristics mainly include complications with impaired social interaction/communication and stereotyped behavior [1]. Patients with ASD likely suffer from other developmental disorders or mental disorders, such as mental retardation, attention deficit hyperactivity disorder, depression, anxiety, and obsessive-compulsive disorder [2,3]. According to the Centers for Disease Control and Prevention, the prevalence of ASD increased from 1.85% in 2020 to 2.77% in 2023 [4,5]. Other nations, including Korea, Germany, and Omani, also reported a considerable increase in the prevalence of ASD [6-8]. However, the etiology and pathogenesis of ASD remain unknown, and early diagnosis and effective treatment are insufficient, resulting in a poor prognosis for most children with ASD. Consequently, most individuals with ASD cannot live, study, and work independently, causing a heavy burden on their families and society [9].

Insulin-like growth factor 1 (IGF-1), a 7.5 kDa peptide hormone found in almost all tissues, is essential for cell growth, differentiation, glucose and uric acid metabolism, and other biological processes [10-14]. It is primarily involved in fetal development [15] and the growth and metabolic regulation of various tissues and organs, including the brain [16], cardiovascular system [17], bone [18], muscle [19], eye [20], uterine [21], and testis [22]. Patients with congenital IGF-1 deficiency exhibit dwarfism and obesity [23]. Increased IGF-1 levels are related to precocious puberty [24], polycystic ovary syndrome [25], and acne [26]. Several single nucleotide polymorphisms (SNPs) of IGF-1 have been observed in non-alcoholic fatty liver disease [27]. Abnormal IGF-1 activation and downstream signaling also contribute to various types of malignancies and treatment resistance [28,29].

IGF-1 functions as a neurotrophic factor in the nervous system and helps in the development and homeostasis of the central nervous system [30]. Abnormal IGF-1 levels in Alzheimer's and Parkinson's diseases may be related to brain cell apoptosis, neuroinflammation, and altered synaptic function [31]. IGF-1 levels change in brain tissue and several types of body fluids of children with ASD [32-34]. A de novo missense mutation of IGF-1 is discovered in children who have ASD with language regression and brain hypometabolism [35]. Therefore, IGF-1 is probably involved in ASD. Maternal immune activation (MIA) and perinatal hypoxia, which are potential causes of ASD [36,37], have been shown to reduce serum IGF-1 levels in mouse offspring [38-40]. Furthermore, IGF-1 treatment enhances neuronal development [41], neuron proliferation [42], and equilibrium of excitatory and inhibitory circuits in brain development [43]; it also elicits anti-inflammatory and antioxidant effects on multiple ASD models related to genetic or environment exposure [44,45]. Therefore, IGF-1 improves ASD-related pathological changes. Indeed, trofinetide (DAYBUE™), an oral small molecule synthetic analog of an N-terminal tripeptide derivative of IGF-1, has been approved by the US Food and Drug Administration (FDA) as the first drug for the treatment of Rett syndrome (RTT) to improve social communication [46]. It provides evidence of the therapeutic role of IGF-1 in the same core symptoms with ASD. In this review, we summarize the changes in IGF-1 levels in patients with ASD, the proposed causes for these changes, and current findings on IGF-1 therapy to offer a treatment option for patients with ASD.

## Review

Biological role of IGF-1 in the brain

Production of IGF-1 in the Brain

IGF-1 is primarily regulated by growth hormone (GH) and is secreted by almost all tissues in the body. The liver synthesizes approximately 70% of the IGF-1 in the circulation after birth. Serum IGF-1 levels steadily increase with age but decline after puberty. IGF-1 expression in the brain is primarily determined through two mechanisms: (1) autocrine/paracrine signaling, where the majority of IGF-1 is produced locally by various cell types in the brain. This production occurs in a diffuse pattern throughout the cerebral cortex, hippocampus, cerebellum, brainstem, and other regions. (2) Endocrine signaling occurs when circulating IGF-1 enters the brain by crossing the blood-brain barrier and the blood-cerebrospinal fluid (CSF) barrier. Thus, increasing the levels of circulating IGF-1 may lead to an elevation in CSF levels [47,48].

Regulation of IGF-1 Activity

High-affinity IGF-binding proteins (IGFBPs) regulate the bioavailability of IGF-1 during embryonic and postnatal development. Six members of the IGFBP family (IGFBP1-IGFBP6) have been identified as carrier proteins that bind to nearly 98% of circulating IGF-1 and extend its half-life. IGFBP3 binds approximately 80% of IGF-1 and, together with the acid-labile subunit, forms a reservoir of IGF-1 in circulation [49]. In addition, except for IGFBP-1, all IGFBPs can be expressed in the brain [50]. IGFBPs hydrolase-mediated proteolytic cleavage, as well as phosphorylation of binding proteins, both reduce the activity of IGFBPs and release free IGF-1. The free IGF-1 then binds to the cell surface glycoprotein insulin-like growth factor-1 receptor (IGF-1R), leading to the activation of the receptor tyrosine kinase. This activation, in turn, phosphorylates insulin receptor substrates (IRS-1-IRS-4) and Src homology collagen (Shc) proteins, thereby activating signal pathways such as the phosphatidylinositol 3-kinase (PI3K)/protein kinase B (AKT) and extracellular signal-regulated kinase (ERK) pathways [51]. These pathways are essential for regulating the cell cycle, autophagy, apoptosis, and protein synthesis [52].

The Role of IGF-1 in Central Nervous System Development

**Figure 1:**
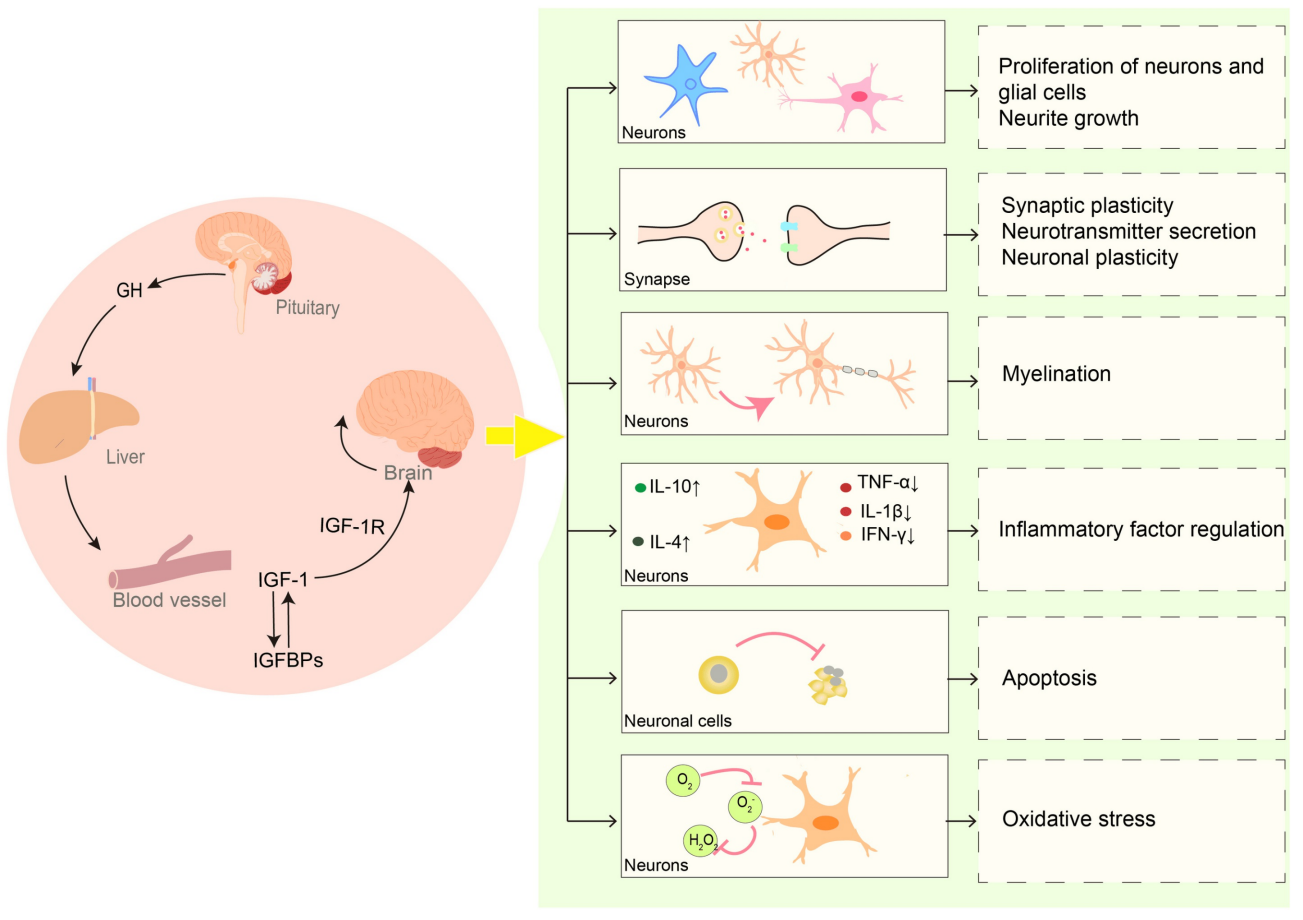
Insulin-like growth factor 1 (IGF-1) plays a role in brain development. IGF-1 regulates the growth and development of the brain through both endocrine and autocrine/paracrine modes. During brain growth and development, IGF-1 promotes the proliferation of neurons and glial cells, as well as the growth of neurites. It also has an impact on the production of neurotransmitters and neuroplasticity. Furthermore, IGF-1 promotes the formation of myelin while suppressing neuroinflammation, apoptosis, and oxidative stress [16,56,57]. The figure was created using Adobe Illustrator.

Previous studies have found that both IGF-1 and IGF-1R are expressed in the central nervous system throughout life, starting from the prenatal stage [52]. IGF-1 mRNA can be detected in almost every region of the brain, especially in areas that have a high concentration of neurons, such as the cerebral cortex and the hippocampus [50]. Similarly, IGF-1R is widely expressed in the brain, and its levels change during different developmental stages. IGF-1R levels in the fetal brain, for instance, are 4-10 times higher than those in adults [53]. Mice lacking IGF-1 or IGF-1R possess a smaller brain capacity and fewer neurons, as well as less myelin and dendrite formation, compared to the control [54]. Serum IGF-1 levels were independently positively linked with total brain, white matter, gray matter, and cerebellar volume in extremely preterm infants born fewer than 28 weeks gestational age [55]. It has been reported that IGF-1 may play a vital role in early brain development (Figure 1). IGF-1 is involved in neuron proliferation and neurite growth. It can assist in the proliferation and differentiation of oligodendrocytes, which promotes myelin formation. In addition, IGF-1 can influence synaptic functional plasticity, enhance neurotransmitter production, and promote the activity of brain-derived neurotrophic factor (BDNF) to impact neuroplasticity [16]. Both in vivo and in vitro, IGF-1 performs a neuroprotective role in brain injury by inhibiting the production of proinflammatory factors, resisting apoptosis, and reducing levels of reactive oxygen species (ROS) [56,57].

Abnormal IGF-1 levels in ASD

As IGF-1 plays a role in neurodevelopmental disorders, a growing number of studies have investigated whether there are alterations in IGF-1 levels in children with ASD. IGF-1 levels in brain tissues and bodily fluids, such as CSF, peripheral blood, and urine, are significantly different in children with ASD compared to non-ASD controls (Table 1).

**Table 1:** Abnormal insulin-like growth factor 1 (IGF-1) levels in autism spectrum disorder (ASD). Refs. [32-34,58-63]

Sample type	Age (mean/mean ±S.D.) (years)		IGF-1 levels in ASD compared to control	Correlation between IGF-1 levels and symptoms	Ref.
	ASD	Control			
Brain tissue	5-44 (-)	5-46 (-)	Upregulated in the anterior cingulate gyrus; no significant difference in the middle frontal gyrus and cerebellum	-	[32]
Brain tissue	2-56 (19.3 ± 3.9)	7.7-56 (27.5 ± 4.1)	No significant difference in the fusiform gyrus	-	[58]
CSF	1.9-6.5 (3.8 ± 1.1)	1.9-5.5 (3.8 ± 1.3)	Downregulated in children under 5 years old	No correlation between IGF-1 levels and head circumference	[59]
CSF	2-16 (-)	1-15 (-)	Downregulated in children under 5 years old; no significant difference in children over 5 years old	Positive correlation between IGF-1 levels and head circumference	[60]
Serum	5-15 (9.5 ± 0.6)	5-15 (8.7 ± 0.5)	Upregulated in serum	Positive correlation between IGF-1 levels and CARS scores	[33]
Serum	4–12 (7.0 ± 2.6)	4–12 (7.8 ± 2.1)	Upregulated in serum	No correlation between serum IGF-1 levels and clinical scale scores (CARS total scores and ABC subscale scores)	[61]
Serum	2–7 (-)	2–7 (-)	Upregulated in serum	Positive correlation between IGF-1 levels and head circumference	[62]
Serum	4–8 (-)	4–8 (-)	Downregulated in children aged 2-3 years; downregulated in children with severe ASD than in children with mild-moderate ASD	Negative correlation between IGF-1 levels and CARS scores	[63]
Urine	2–5 (3.1 ± 0.9)	2–5 (3.3 ± 1.2)	Downregulated in urine	-	[34]

Abnormal IGF-1 Levels in the Brains of Patients With ASD

IGF-1 levels are significantly elevated in the anterior cingulate gyrus of postmortem brain tissues from ASD patients, but not in the middle frontal gyrus, fusiform gyrus, and cerebellum [32,58]. IGF-1 levels in the CSF are significantly lower in ASD patients under the age of five years when compared to non-ASD controls [59,60]. However, there is no significant change in IGF-1 levels in the CSF in ASD patients beyond the age of five years [60].

Aberrant IGF-1 Levels in the Peripheral Blood of Patients With ASD

There are conflicting results regarding the levels of IGF-1 in peripheral blood. Some studies have reported that serum IGF-1 levels in children with ASD were significantly higher than in typically developing children [33,61,62]. However, one analysis of the IGF-1 (rs12579108) promoter polymorphism has found that children with the AA genotype showed decreased serum IGF-1 concentrations and a higher risk of ASD [64]. This implies that decreased IGF-1 serum levels may be associated with the development of ASD. Indeed, a recent study discovered that serum IGF-1 levels in children with ASD aged two to three years were lower than those in the healthy group. Further, children with severe ASD showed significantly lower serum levels of IGF-1 and IGFBP-3 compared to children with mild-to-moderate ASD. The serum levels of IGF-1 and IGFBP-3 were negatively correlated with the total score of CARS, indicating a connection to the severity of ASD. However, no significant differences in serum IGF-1 and IGFBP-3 levels were found between children aged three to seven years and the control group [63]. These results suggest that IGF-1 may be a potential serum marker in toddlers with ASD.

Abnormal IGF-1 Levels in the Urine of Patients With ASD

Previous research has proposed that free IGF-1 in peripheral blood may be the main source of IGF-1 in urine [65]. Thus, urine IGF-1 levels may reflect serum IGF-1 levels that are activated. One study has reported that children with ASD aged two to five years showed lower levels of IGF-1 and IGFBP-3 in urine [34], which is consistent with the findings of Li et al. in serum and Riikonen et al. in CSF [60,63]. The above results suggest that IGF-1 might serve as a marker for bodily fluids in preschool-age children with ASD.

Potential mechanisms of decreased IGF-1 levels in ASD

The reason for the decrease in body fluid IGF-1 levels in preschool children with ASD is unclear. However, a recent study has found that a specific IGF-1 SNP in children with ASD may impact IGF-1 levels [64]. In addition, risk factors for ASD such as maternal immune activation (MIA) and perinatal hypoxia may be linked to reduced IGF-1 levels in the offspring.

The IGF-1 SNP May Decrease the Levels of IGF-1 in the Serum

A recent study investigated the association between the IGF-1 (rs12579108) promoter polymorphism and its serum concentration with ASD, using 200 ASD patients and 198 normal controls. They reported that among three genotypes (AA, CA, CC) in the IGF-1 (rs12579108) promoter, the incidence of the AA genotype was significantly higher in ASD patients (11%) compared to controls (4.5%). The allele A was linked to an elevated risk of ASD. Among the three genotypes, patients with the AA genotype exhibited the lowest serum IGF-1 concentrations. Although serum IGF-1 concentrations were also the lowest in the control group with the AA genotype, they were even lower in the ASD group with the AA genotype compared to the control group with the AA genotype. This suggests that the AA genotype is associated with lower IGF-1 serum levels and may act as a risk factor for ASD [64].

In addition, apart from reducing IGF-1 expression, some mutations may cause a functional defect in IGF-1. Using whole exome sequencing in 100 children with ASD and their unaffected parents, researchers identified a de novo missense mutation (c.251 G>A) in the IGF-1 gene in a female patient. This patient experienced a decline in language and intellectual abilities after 18 months, suggesting that a functional deficiency of IGF-1 may also be linked to ASD [35].

MIA Mediates the Suppression of IGF-1 Levels Through IL-6

Previous studies have found that multiple factors inducing MIA are associated with an increased risk of ASD [37]. Offspring of mothers who were MIA exhibit ASD-like behaviors, such as repetitive behavior and social abnormalities [66]. In a mouse model of MIA induced by prenatal infection, there was upregulation of IL-6 and downregulation of IGF-1 expression in the placenta. The expression of IGF-1 was found to be upregulated in the placentas of IL-6 knockout MIA mice [38], indicating that upregulated IL-6 is responsible for the suppression of IGF-1 expression. Additionally, IL-6 treatment facilitated the binding of SOCS3 to IRS-1 or IGF-1R, leading to the suppression of IGF-1 activation [67]. Interestingly, a simultaneous increase in the expression of SOCS3 and IL-6 was observed in the placentas of MIA mice [38]. Therefore, the decreased levels of IGF-1 in the MIA model may be attributed to the inhibitory effect of IL-6 through SOCS3.

Perinatal Hypoxia May Reduce Serum IGF-1 Levels

Perinatal hypoxia has been proposed to be associated with an increased risk of ASD [36]. In prospective studies of newborns with perinatal hypoxic-ischemia encephalopathy (HIE) and healthy newborns, the mean serum IGF-1 levels are significantly decreased in the HIE group. Furthermore, IGF-1 levels were lower in severe HIE compared to mild HIE. The level rose as the patients showed clinical improvement in the following days [68,69]. Mice with HIE induced by perinatal hypoxia exhibited social deficits similar to those seen in individuals with ASD [70]. In both the acute (24-hour) and subacute (15-day) phases of perinatal hypoxic-ischemia in mice, serum IGF-1 levels were reduced [40]. Therefore, the reduced levels of IGF-1 in some ASD patients may be attributed to perinatal hypoxia. However, the mechanism is still unclear.

IGF-1 improves changes in multiple ASD models

ASD is highly genetically heterogeneous, with over 100 identified risk genes that play diverse roles in epigenetic regulation and synaptic function. On the other hand, environmental risk factors are also receiving increasing attention due to their pervasive effect and their ability to influence the early phases of fetal development [71]. In various genetic forms of ASD models and ASD models associated with environmental risk factors, IGF-1 treatment has been shown to improve core symptoms or pathological changes related to ASD.

IGF-1 Enhances Neuronal Development

To obtain ASD-specific expression profiles, the authors performed RNA-sequencing on neurons derived from eight patients with ASD and seven neurotypical individuals. They identified 345 differentially expressed genes between ASD and control groups. After treatment with recombinant human IGF-1, 124 genes in ASD neurons were restored to a neurotypical level. These genes were found to be enriched within synapse genes Linker, Mendes, and Marchetto [72], indicating that IGF-1 can potentially restore synapse development or function in ASD in vitro.

The scaffold protein SH3 and multiple ankyrin repeat domains 2 (SHANK2) act as a protein anchor at the postsynaptic density. ASD is associated with SHANK2 heterozygous loss-of-function mutations [73,74]. Neurons differentiated from human-induced pluripotent stem cells (iPSCs) with SHANK2 knockdown exhibit shorter dendritic length and fewer neurite arborizations in glutamatergic neurons. After plating the neurons, IGF-1 therapy significantly improves both dendritic length and neurite arborization defects in SHANK2 knockdown excitatory neurons. However, no significant difference in neurite arborization was observed in the control group, with or without IGF1 therapy [75]. These findings imply that the IGF-1 signal plays a crucial role in correcting morphological deficiencies in glutamatergic neurons with SHANK2 loss.

Loss-of-function mutations in dual specificity tyrosine phosphorylation-regulated kinase 1A (DYRK1A) have been reported to be associated with ASD [76]. DYRK1A conditional heterozygous (cHet) mice exhibit ASD-like behaviors, such as social abnormalities and repetitive behavior, as well as microcephaly. A significant decrease in soma size and basal dendritic branching is found in layer V pyramidal neurons in the medial prefrontal cortex (mPFC) of the mouse model. Phosphorylation levels of S6, which serve as a readout of mTORC1 activity, are also reduced in layer V pyramidal neurons, indicating a decrease in the transduction of the mTOR signaling pathway. In addition, the authors have identified BDNF, mTOR, and IGF-1 as potential upstream regulators of the altered proteome in DYRK1A cHet mice. (1-3) IGF-1, a tripeptide cleaved from the N-terminus of IGF-1, treatment can rescue the decreased cortical mass, soma size, and phosphorylated S6 in layer V neurons [77]. These studies suggest that IGF-1 may enhance neuronal morphology through mTOR signaling in mice with DYRK1A deficits.

The Fragile X messenger ribonucleoprotein 1 (FMR1) gene mutations have been extensively documented to be linked with ASD phenotypes [78]. FMR1 knockout mice exhibit hyperactivity, anxiety, deficits in learning and memory, and abnormal social recognition. Furthermore, FMR1 knockout mice exhibit elevated levels of phosphorylated ERK and phosphorylated Akt in their brain and peripheral blood lymphocytes. Spine density is significantly increased in FMR1 knockout hippocampus primary cell culture [41]. NNZ-2566, a novel analog of (1-3) IGF-1, improved social behavior, learning, and memory impairments in FMR1 knockout mice. Further study revealed that (1-3) IGF-1 supplementation reversed the increased levels of phosphorylated ERK and Akt protein expression in the brain and lymphocytes, as well as the increased spine density in the hippocampus of FMR1 knockout mice [41]. These results suggest that IGF-1 may compensate for the abnormal signaling pathway and hippocampal neuronal development disrupted by FMR1 gene mutations.

MECP2 is also a risk gene for ASD [79]. A whole-exome sequencing study of 120 patients with ASD identified three missense mutations in MECP2: p.P152L (c.455C>T), p.P376S (c.1162C>T), and p.R294X (c.880C>T). These missense mutations caused the loss of function of MECP2 [80]. Using gene editing to generate human embryonic stem cells (ESCs) with a MECP2 allele that has a loss of function, researchers found that the rates of neurons (including excitatory and inhibitory neurons) and glial cells derived from mutant human ESCs remained unchanged. However, the size of the soma and nucleus of neurons, particularly inhibitory neurons, as well as the branching of neurites, were all reduced [81]. In addition, neurons with MECP2 mutations exhibited reduced levels of phosphorylated AKT and S6, as well as decreased total RNA levels and protein synthesis. IGF-1 treatment can enhance phosphorylated AKT and S6, as well as neuronal soma size, neurite arborization, and protein synthesis in MECP2 mutant neurons, similar to the effects of PTEN deletion (a negative regulator of PI3K) [81]. These studies suggest that IGF-1 may play a role in neuronal development and protein synthesis through PTEN/PI3K. In a mouse model with MECP2 deficiency, reduced lifespan, decreased brain weight, and impaired excitatory synaptic transmission have been observed. Notably, the serum level of IGF-1 protein is also decreased in MECP2-deficient mice. Further study revealed that treatment of MECP2-deficient mice with (1-3) IGF-1 from two weeks of age can improve survival in mice, brain structure, increase brain weight, PSD-95 (a postsynaptic scaffold protein at excitatory synapses) levels, and spine density of layer V pyramidal neurons. Furthermore, the reduction in the excitatory postsynaptic current (EPSC) amplitude was partially reversed by treatment with (1-3) IGF-1 [82]. These studies support the involvement of IGF-1 in neuronal development and synaptic function.

IGF-1 Promotes the Survival of Neurons

GRB10 interacting GYF protein 1 (GIGYF1) is a high-confidence ASD risk gene [83]. GIGYF1 cKO mice display repetitive and stereotyped behaviors, anxiety, and learning and memory deficits. GIGYF1 cKO mice have a decreased number of upper-cortical neurons, which can be attributed to the diminished proliferation of neural progenitor cells (NPCs) due to the cell cycle remaining in the S phase. Furthermore, GIGYF1-deficient mice exhibit disruption of the IGF-1R/ERK pathway during early brain development. In GIGYF1 KO HEK293T cells, disruption of IGF-1R recycling from the nucleus to the cell membrane is observed, which also leads to disruption of the IGF-1R/ERK pathway. It was discovered that treating neurospheres from GIGYF1-deficient embryonic mice at E14.5 with IGF-1 can enhance neurosphere proliferation and improve the impaired proliferation of NPC in the neurosphere using neurosphere formation assays [83]. This suggests that IGF-1 therapy may promote neuron proliferation during the embryonic period.

The EBF transcription factor 2 (EBF2) plays a crucial role in cerebellar development, especially in controlling the development and survival of Purkinje cells [84]. The number of Purkinje cells is reduced by 38% through apoptosis in EBF2 null mutant mice [85]. In EBF2-null mice, the levels of IGF-1 mRNA and protein, as well as its downstream phosphorylated AKT (Thr308 and Ser473) and GSK3β (Ser9), are decreased in the cerebellum and Purkinje cells. Further study has found that EBF2 can bind directly to the IGF-1 promoter and activate IGF1 expression. These results support the suppression of the IGF-1/PI3K/AKT pathway in EBF2 null mice. In addition, H-1356, a competitive IGF1R inhibitor, increases the apoptosis of Purkinje cells in wild-type slices, while exogenous IGF1 treatment significantly reduces the apoptosis of Purkinje cells in EBF2 null cerebellar slices [86]. These studies confirm the crucial role of IGF-1 in the survival of Purkinje cells.

One study discovered that IGF-1 promotes cell survival and proliferation while decreasing the expression of G protein-coupled receptor 17 (GPR17) and FOXO1 in SK-N-SH cells, a cell line model for ASD. GPR17 is a P2Y-like receptor that has been reported to mediate neuronal injury and microglia proliferation following focal cerebral ischemia in rats [87]. Antagonizing GPR17 and inhibiting FOXO1 both played a role comparable to IGF-1. Blocking PI3K signaling with LY294002 prevented the impact of IGF-1 on GPR17 suppression. Moreover, IGF-1 led to an increase in FOXO1 nuclear export and a decrease in FOXO1 binding to the GPR17 promoter in SK-N-SH cells. As a result, IGF-1 may improve cell survival and proliferation in SK-N-SH cells by facilitating the nuclear export of FOXO1 and reducing its binding to the GPR17 promoter via PI3K/Akt signaling [88].

In addition to genetic models of ASD, IGF-1 treatment can protect neurons from apoptosis in ASD models produced by environmental factors. Subcutaneous administration of IGF-1 at 24 and 48 hours following hypoxic ischemia can decrease brain atrophy and improve long-term memory and cognitive function in rats. In the cortex of newborn rats, neurons deprived of oxygen and glucose showed a significant reduction in neuronal apoptosis after 24 hours of treatment with IGF-1 [89].

IGF-1 Influences the Balance of Synaptic Excitation and Inhibition

The synaptic excitation-to-inhibition ratio refers to the relative contributions of excitatory and inhibitory synaptic inputs. The ratio of synaptic excitation to inhibition is altered in individuals with ASD who have distinct genetic forms. Some studies have reported a greater decrease in inhibition than excitation [90], while others have reported a greater decrease in excitation than inhibition [91,92], which may cause dysregulation of synapse and circuit excitability. Marchetto et al. reprogrammed skin fibroblasts from ASD patients with early brain overgrowth and non-ASD controls with normal brain size, to generate iPSCs, NPCs, and neurons. When recombinant human IGF-1 was added during the differentiation process, the number of GABAergic neurons increased in all cells derived from individuals with ASD. After differentiation, mature ASD-derived cultures treated with IGF-1 displayed an inclination to improve neuronal spike quantity, suggesting that IGF-1 may partially restore network establishment in ASD-derived neuronal cultures [93].

IGF-1 treatment can also correct impairments in hippocampal alpha-amino-3-hydroxy-5-methyl-4-isoxazole propionic acid (AMPA) signaling, long-term potentiation (LTP), and motor function in autism mouse models with SHANK3 deficiency. This indicates that IGF1 can restore postsynaptic excitability [94]. Further research has revealed that IGF-1 suppresses N-methyl-D-aspartate (NMDA)-induced excitotoxicity via IRS2, resulting in neuroprotection. In turn, the excitatory neurotransmitter glutamate reduces IGF-1R phosphorylation, disrupting IGF-1 signaling. Additionally, IGF-1-induced phosphorylation of GSK3β regulates the connection between N-methyl-D-aspartate receptor (NMDAR)-dependent LTP and long-term depression (LTD), which are the primary forms of enduring synaptic strength changes in the brain [43]. These results imply the role of IGF-1in regulating the E-I ratio. However, further attention and effort are needed to clarify its mechanism in ASD.

IGF-1 Regulates Neuroinflammation

Multiple studies have reported unregulated inflammation in patients with ASD in the absence of insult or injury. Sustained activation of microglia and astrocytes contributes to neural deficits in ASD, which may disrupt neural connectivity and contribute to core symptoms in ASD [95,96]. Since MIA inhibits IGF-1 levels in the placenta through increased IL-6, it is worth exploring the role of IGF-1 in the neuroinflammation in ASD. In a mouse model of ASD with a SHANK3 gene mutation, hyperbaric oxygen therapy dramatically increases the expression levels of IGF-1 and decreases the number of microglia expressing ionized calcium-binding adapter molecule 1 (Iba1), a marker of microglia activation. This suggests a negative correlation between IGF-1 levels and microglia activation [97]. Injection of propionic acid (PPA) into rats is a commonly used animal model for ASD. IGF-1 levels in brain homogenate and CSF were significantly reduced in ASD mice model induced with PPA. After 4-hydroxyisoleucine therapy, increased levels of IGF-1 and decreased levels of inflammatory cytokines (TNF-α and IL-1β) were observed in the plasma and brain homogenate of the PPA-induced ASD animal model. It indirectly suggests a link between IGF-1 and neuroinflammation [98]. Substantial evidence has shown that IGF-1 signaling reduces neuroinflammation by inhibiting glial activation and cytokine levels, such as IL-1β, IL-6, IL-10, and TNF-α [99-101]. As a result, investigating the role of IGF-1 in ASD with dysregulated neuroinflammation driven by different etiologies may provide potential therapeutics for ASD.

IGF-1 Protects Against Oxidative Stress

An imbalance between pro-oxidants and antioxidants causes oxidative stress, which triggers the attack of cells by ROS or reactive nitrogen species (RNS). When ROS accumulate within cells and are not properly scavenged, they can cause oxidative stress. Superoxide (O2−) is formed during normal oxygen metabolism and is quickly transformed into hydrogen peroxide (H2O2) by superoxide dismutases (SODs) [102]. O2− and H2O2 are both harmful to cells. Some pathways, such as catalase and glutathione peroxidase (GPx), can convert H2O2 to H2O. Free reduced glutathione (GSH), an antioxidant with cytoprotective effects, donates an electron to H2O2 and is converted to the oxidized form GSSG through the catalytic action of GPx. GSH can be replenished through the action of GSH reductase (GR) [102]. In recent years, several groups have reviewed the neuropathology of ASD concerning oxidative stress, which includes mitochondrial dysfunction, decreased antioxidant enzyme activity, and increased lipid peroxidation [103-105]. In addition, due to their low cellular GSH concentrations and poor GSH redox capacity, children with ASD are more vulnerable to oxidative stress [106].

IGF-1-deficient mice exhibit increased expression of pro-oxidant genes (e.g., prostaglandin-endoperoxide synthase 2, nitric oxide synthase 1), suppressed expression of antioxidant genes (e.g., GPx 1 and 8, catalase, superoxide dismutase 2), and elevated levels of lipid peroxidation markers. Brian’s oxidative damage is mitigated by IGF-1 treatment [45]. IGF-1 also boosts GSH synthesis in neuroblastoma SH-SY5Y cells (a cell line model for ASD) by upregulating the expression of the glutamate-cysteine ligase modifier subunit (GCLM), which is mediated by increased nuclear factor erythroid 2-related factor 2 (Nrf2) protein in the nucleus. Interestingly, a PI3K inhibitor abrogated the IGF-1-induced upregulation of GCLM [107]. Furthermore, inhibition of IGFR elevated ROS levels induced by glutamate stimulation in both astrocytes and cerebrovascular endothelial cells [108], demonstrating that IGF-1 signaling contributes to astrocyte protection by inhibiting ROS levels. Furthermore, the accumulation of ROS can lead to the production of oxidized proteins and lipid peroxidation, which may directly increase neuroinflammation in ASD [102]. Hence, it is important to investigate whether IGF-1 is involved in the interaction between oxidative stress and neuroinflammation in ASD.

**Figure 2:**
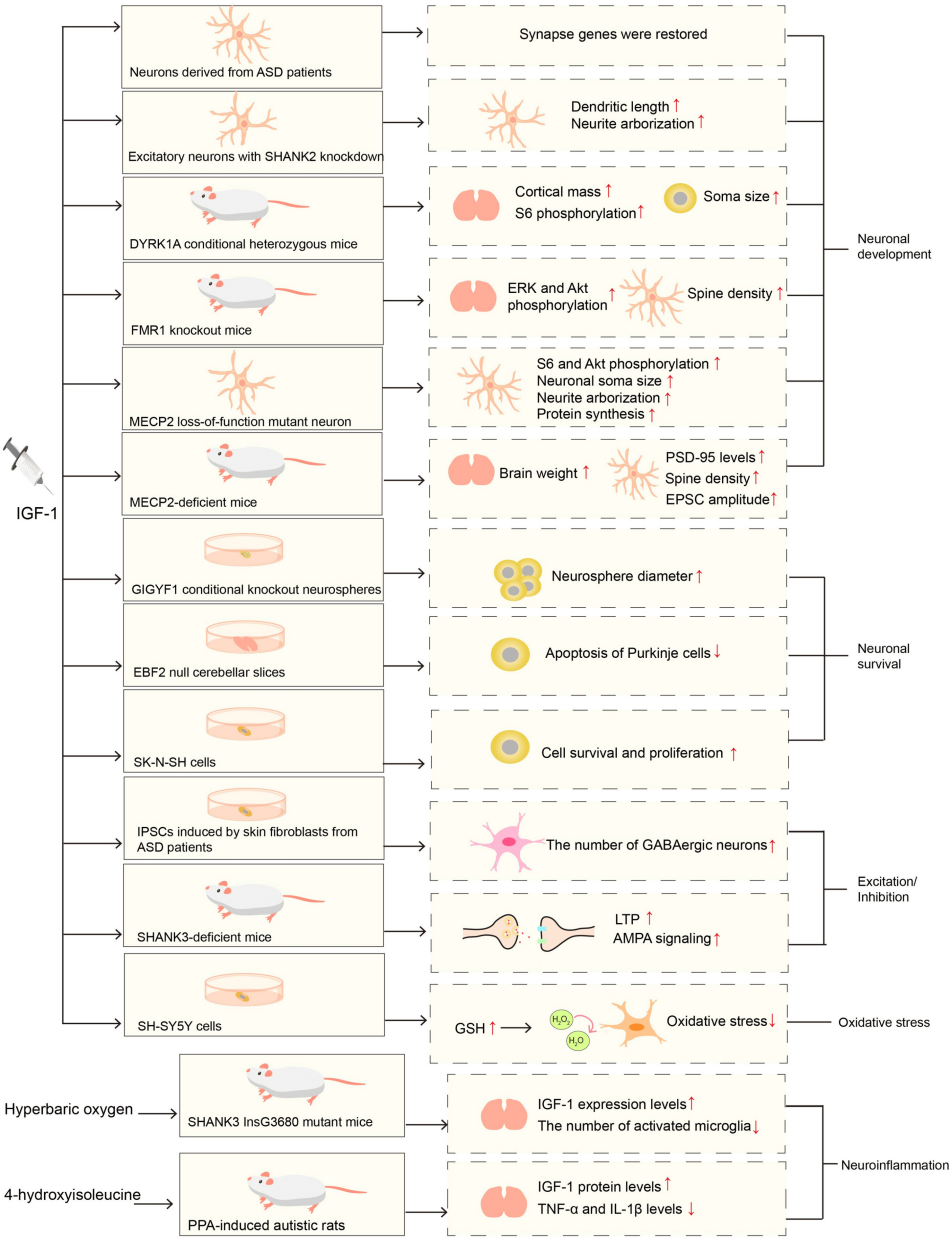
Treatment with insulin-like growth factor 1 (IGF-1) improves pathological changes in multiple models of autism spectrum disorder (ASD). IGF-1 enhances neuronal development in neurons derived from individuals with ASD and in genetic-associated ASD mice, such as those with DYRK1A deficit, FMR1 knockout, and MECP2 deficit. IGF-1 promotes the survival of neurons in GIGYF1 conditional knockout neurospheres, EBF2 null cerebellar slices, and the SK-N-SH cell line. IGF-1 enhances the balance of synaptic excitation and inhibition in induced pluripotent stem cells (iPSCs) derived from skin fibroblasts of individuals with ASD and SHANK3-deficient mice. IGF-1 protects against oxidative stress in the SH-SY5Y cell line. IGF-1 may also regulate neuroinflammation directly or indirectly. This is evidenced by the increased IGF-1 and decreased microglia activation or cytokine levels observed in both hyperbaric oxygen treatment in SHANK3 mutant mice and 4-hydroxyisoleucine therapy in propionic acid (PPA)-induced ASD rats [41,72,75,77,81-83,86,88,93,94,97,98,107]. The figure was created using Adobe Illustrator.

These improvements encompass neuronal development, neural cell survival and proliferation, the balance of excitation and inhibition in brain circuits, neuroimmunology, and the status of oxidative stress (Figure 2).

Application of IGF-1 in the treatment of neurodevelopmental disorders with ASD-like core symptoms

Recombinant IGF-1 (mecasermin) was approved for the treatment of GH insensitivity (GHI) syndrome or IGF-1 deficiency (IGFD) by the US FDA in 2005 [109]. Because of IGF-1’s function in brain development [110] and its ability to cross the blood-brain barrier, it has emerged as a viable therapeutic method for neurodevelopmental disorders [111]. In the last decade, numerous studies have indicated IGF-1’s efficacy in rescuing neurodevelopmental deficits in diseases with ASD-like phenotypes, especially in RTT [77,78,112]. We are thrilled to learn that the FDA-approved trofinetide (DAYBUE™), an oral small molecule synthetic analog of glycine-proline-glutamate (GPE), an N-terminal tripeptide derivative of IGF-1, as the first drug for the treatment of RTT in adult and pediatric patients two years of age and older in March 2023 [46].

In RTT patients, four weeks of incremental dose treatment with mecasermin increased IGF-1 levels in both peripheral blood and CSF, as well as improved anxiety and respiratory function [113]. Trofinetide therapy resulted in clinically relevant improvements in a phase 2 randomized investigation of 82 patients with RTT aged 5 to 15 [114]. In a recent randomized phase 3 study, females aged 5 to 20 with RTT were given twice-daily oral trofinetide (n = 93) or placebo (n = 94) for 12 weeks, which improved key symptoms, including communication capacity [112].

Phelan-McDermid syndrome (PMS) is characterized by general developmental delays, deficiencies in verbal or motor skills, and ASD-like behaviors due to a heterozygous deletion of chromosome 22q13.3 coupled with SHANK3 deletion or mutation [115]. A clinical trial of nine PMS kids aged 5-15 years has found that IGF-1 treatment was safe and resulted in significant improvements in social deficits and repetitive behaviors [116].

Fragile X syndrome (FXS) is the most common single-gene disorder associated with ASD, exhibiting phenotypic features such as ASD-like behaviors and cognitive abnormalities [117]. In a phase 2 randomized study, trofinetide at a dose of 70 mg/kg demonstrated improvements in social avoidance, anxiety, communication, repetitive behaviors, hyperactivity, motor impairments, and sensory sensitivity. Furthermore, there were no reported deaths or serious adverse events during trofinetide treatment, indicating initial evidence of the safety of trofinetide therapy [118]. These studies indicate that IGF-1 can alleviate core symptoms of ASD.

## Conclusions

Because of the reduced levels of IGF-1 in human body fluids of preschool-age ASD patients, as well as the correlation between serum IGF-1 level and ASD core symptoms, IGF-1 has the potential to be a molecular marker of ASD to assist in its early diagnosis. Further research may broaden our application of IGF-1 as a molecular marker. Since the FDA recently approved IGF-1 as the sole drug for RTT for partially improving ASD-like core disorders, it may be beneficial in the treatment of ASD. More academic and clinical communities could prioritize IGF-1 research in ASD treatment. In fact, a clinical trial to investigate the efficacy of IGF-1 in ASD individuals is presently underway [ClinicalTrials.gov identifier: NCT01970345]. The outcomes are fundamental in identifying new ASD interventions.

## References

[1] Sharma SR, Gonda X, Tarazi FI (2018). Autism spectrum disorder: classification, diagnosis and therapy. Pharmacol Ther.

[2] Elliott SJ, Marshall D, Morley K, Uphoff E, Kumar M, Meader N (2021). Behavioural and cognitive behavioural therapy for obsessive compulsive disorder (OCD) in individuals with autism spectrum disorder (ASD). Cochrane Database Syst Rev.

[3] Hirota T, King BH (2023). Autism spectrum disorder: a review. JAMA.

[4] Maenner MJ, Shaw KA, Baio J (2020). Prevalence of autism spectrum disorder among children aged 8 years - Autism and Developmental Disabilities Monitoring Network, 11 sites, United States, 2016. MMWR Surveill Summ.

[5] Maenner MJ, Warren Z, Williams AR (2023). Prevalence and characteristics of autism spectrum disorder among children aged 8 years - Autism and Developmental Disabilities Monitoring Network, 11 sites, United States, 2020. MMWR Surveill Summ.

[6] Yoo SM, Kim KN, Kang S, Kim HJ, Yun J, Lee JY (2022). Prevalence and premature mortality statistics of autism spectrum disorder among children in Korea: a nationwide population-based birth cohort study. J Korean Med Sci.

[7] Bachmann CJ, Gerste B, Hoffmann F (2018). Diagnoses of autism spectrum disorders in Germany: time trends in administrative prevalence and diagnostic stability. Autism.

[8] Al-Mamri W, Idris AB, Dakak S (2019). Revisiting the prevalence of autism spectrum disorder among Omani children: a multicentre study. Sultan Qaboos Univ Med J.

[9] Rogge N, Janssen J (2019). The economic costs of autism spectrum disorder: a literature review. J Autism Dev Disord.

[10] Al-Samerria S, Radovick S (2021). The role of insulin-like growth factor-1 (IGF-1) in the control of neuroendocrine regulation of growth. Cells.

[11] Clemmons DR (2006). Involvement of insulin-like growth factor-I in the control of glucose homeostasis. Curr Opin Pharmacol.

[12] Allan GJ, Flint DJ, Patel K (2001). Insulin-like growth factor axis during embryonic development. Reproduction.

[13] Mandal AK, Leask MP, Sumpter NA, Choi HK, Merriman TR, Mount DB (2023). Genetic and physiological effects of insulin-like growth factor-1 (IGF-1) on human urate homeostasis. J Am Soc Nephrol.

[14] Cohick WS, Clemmons DR (1993). The insulin-like growth factors. Annu Rev Physiol.

[15] Hellström A, Ley D, Hansen-Pupp I (2016). Insulin-like growth factor 1 has multisystem effects on foetal and preterm infant development. Acta Paediatr.

[16] Dyer AH, Vahdatpour C, Sanfeliu A, Tropea D (2016). The role of insulin-like growth factor 1 (IGF-1) in brain development, maturation and neuroplasticity. Neuroscience.

[17] Macvanin M, Gluvic Z, Radovanovic J, Essack M, Gao X, Isenovic ER (2023). New insights on the cardiovascular effects of IGF-1. Front Endocrinol (Lausanne).

[18] Fang J, Zhang X, Chen X (2023). The role of insulin-like growth factor-1 in bone remodeling: A review. Int J Biol Macromol.

[19] Roberston MJ, Raghunathan S, Potaman VN, Zhang F, Stewart MD, McConnell BK, Schwartz RJ (2020). CRISPR-Cas9-induced IGF1 gene activation as a tool for enhancing muscle differentiation via multiple isoform expression. FASEB J.

[20] Truong T, Silkiss RZ (2023). The role of insulin-like growth factor-1 and its receptor in the eye: a review and implications for IGF-1R inhibition. Ophthalmic Plast Reconstr Surg.

[21] Gonzalez A, Berg MD, Southey B, Dean M (2022). Effect of estradiol and IGF1 on glycogen synthesis in bovine uterine epithelial cells. Reproduction.

[22] Cannarella R, Crafa A, La Vignera S, Condorelli RA, Calogero AE (2021). Role of the GH-IGF1 axis on the hypothalamus-pituitary-testicular axis function: lessons from Laron syndrome. Endocr Connect.

[23] Ranke MB, Wölfle J, Schnabel D, Bettendorf M (2009). Treatment of dwarfism with recombinant human insulin-like growth factor-1. Dtsch Arztebl Int.

[24] He J, Kang Y, Zheng L (2023). Correlation of serum levels of LH, IGF-1 and leptin in girls with the development of idiopathic central precocious puberty. Minerva Pediatr (Torino).

[25] Li H, Zhang Y, Liu C, Zhang Y, Yang H, Fu S, Lv H (2023). Association of insulin-like growth factor-1 with polycystic ovarian syndrome: a systematic review and meta-analysis. Endocr Pract.

[26] Albalat W, Darwish H, Abd-Elaal WH, AbouHadeed MH, Essam R (2022). The potential role of insulin-like growth factor 1 in acne vulgaris and its correlation with the clinical response before and after treatment with metformin. J Cosmet Dermatol.

[27] Nobakht H, Mahmoudi T, Rezamand G (2022). Association of RS5742612 polymorphism in the promoter region of IGF1 gene with nonalcoholic fatty liver disease: a case-control study. Lab Med.

[28] Denduluri SK, Idowu O, Wang Z (2015). Insulin-like growth factor (IGF) signaling in tumorigenesis and the development of cancer drug resistance. Genes Dis.

[29] Basu R, Kopchick JJ (2023). GH and IGF1 in cancer therapy resistance. Endocr Relat Cancer.

[30] Torres-Aleman I (2010). Toward a comprehensive neurobiology of IGF-I. Dev Neurobiol.

[31] Riikonen R (2017). Insulin-like growth factors in the pathogenesis of neurological diseases in children. Int J Mol Sci.

[32] Vargas DL, Nascimbene C, Krishnan C, Zimmerman AW, Pardo CA (2005). Neuroglial activation and neuroinflammation in the brain of patients with autism. Ann Neurol.

[33] Robinson-Agramonte ML, Michalski B, Vidal-Martinez B, Hernández LR, Santiesteban MW, Fahnestock M (2022). BDNF, proBDNF and IGF-1 serum levels in naïve and medicated subjects with autism. Sci Rep.

[34] Anlar B, Oktem F, Bakkaloglu B (2007). Urinary epidermal and insulin-like growth factor excretion in autistic children. Neuropediatrics.

[35] Tran KT, Le VS, Bui HT (2020). Genetic landscape of autism spectrum disorder in Vietnamese children. Sci Rep.

[36] Khachadourian V, Mahjani B, Sandin S, Kolevzon A, Buxbaum JD, Reichenberg A, Janecka M (2023). Comorbidities in autism spectrum disorder and their etiologies. Transl Psychiatry.

[37] Kwon HK, Choi GB, Huh JR (2022). Maternal inflammation and its ramifications on fetal neurodevelopment. Trends Immunol.

[38] Hsiao EY, Patterson PH (2011). Activation of the maternal immune system induces endocrine changes in the placenta via IL-6. Brain Behav Immun.

[39] Davenport ML, D'Ercole AJ, Underwood LE (1990). Effect of maternal fasting on fetal growth, serum insulin-like growth factors (IGFs), and tissue IGF messenger ribonucleic acids. Endocrinology.

[40] Kartal Ö, Aydınöz S, Kartal AT (2016). Time dependent impact of perinatal hypoxia on growth hormone, insulin-like growth factor 1 and insulin-like growth factor binding protein-3. Metab Brain Dis.

[41] Deacon RM, Glass L, Snape M, Hurley MJ, Altimiras FJ, Biekofsky RR, Cogram P (2015). NNZ-2566, a novel analog of (1-3) IGF-1, as a potential therapeutic agent for fragile X syndrome. Neuromolecular Med.

[42] Takata S, Sakata-Haga H, Shimada H (2022). Lif-IGF axis contributes to the proliferation of neural progenitor cells in developing rat cerebrum. Int J Mol Sci.

[43] Ge L, Liu S, Rubin L, Lazarovici P, Zheng W (2022). Research progress on neuroprotection of insulin-like growth factor-1 towards glutamate-induced neurotoxicity. Cells.

[44] Labandeira-Garcia JL, Costa-Besada MA, Labandeira CM, Villar-Cheda B, Rodríguez-Perez AI (2017). Insulin-like growth factor-1 and neuroinflammation. Front Aging Neurosci.

[45] Puche JE, Muñoz Ú, García-Magariño M, Sádaba MC, Castilla-Cortázar I (2016). Partial IGF-1 deficiency induces brain oxidative damage and edema, which are ameliorated by replacement therapy. Biofactors.

[46] Harris E (2023). Trofinetide receives FDA approval as first drug for Rett syndrome. JAMA.

[47] Fernandez AM, Torres-Alemán I (2012). The many faces of insulin-like peptide signalling in the brain. Nat Rev Neurosci.

[48] Ashpole NM, Sanders JE, Hodges EL, Yan H, Sonntag WE (2015). Growth hormone, insulin-like growth factor-1 and the aging brain. Exp Gerontol.

[49] Bailes J, Soloviev M (2021). Insulin-like growth factor-1 (IGF-1) and its monitoring in medical diagnostic and in sports. Biomolecules.

[50] Russo VC, Gluckman PD, Feldman EL, Werther GA (2005). The insulin-like growth factor system and its pleiotropic functions in brain. Endocr Rev.

[51] Józefiak A, Larska M, Pomorska-Mól M, Ruszkowski JJ (2021). The IGF-1 signaling pathway in viral infections. Viruses.

[52] Hakuno F, Takahashi SI (2018). IGF1 receptor signaling pathways. J Mol Endocrinol.

[53] Szczęsny E, Slusarczyk J, Głombik K (2013). Possible contribution of IGF-1 to depressive disorder. Pharmacol Rep.

[54] Beck KD, Powell-Braxton L, Widmer HR, Valverde J, Hefti F (1995). Igf1 gene disruption results in reduced brain size, CNS hypomyelination, and loss of hippocampal granule and striatal parvalbumin-containing neurons. Neuron.

[55] Hellström W, Hortensius LM, Löfqvist C (2023). Postnatal serum IGF-1 levels associate with brain volumes at term in extremely preterm infants. Pediatr Res.

[56] Guan J, Bennet L, Gluckman PD, Gunn AJ (2003). Insulin-like growth factor-1 and post-ischemic brain injury. Prog Neurobiol.

[57] Mangiola A, Vigo V, Anile C, De Bonis P, Marziali G, Lofrese G (2015). Role and importance of IGF-1 in traumatic brain injuries. Biomed Res Int.

[58] Cioana M, Michalski B, Fahnestock M (2020). Insulin-like growth factor and insulin-like growth factor receptor expression in human idiopathic autism fusiform gyrus tissue. Autism Res.

[59] Vanhala R, Turpeinen U, Riikonen R (2001). Low levels of insulin-like growth factor-I in cerebrospinal fluid in children with autism. Dev Med Child Neurol.

[60] Riikonen R, Makkonen I, Vanhala R, Turpeinen U, Kuikka J, Kokki H (2006). Cerebrospinal fluid insulin-like growth factors IGF-1 and IGF-2 in infantile autism. Dev Med Child Neurol.

[61] Şimşek F, Işık Ü, Aktepe E, Kılıç F, Şirin FB, Bozkurt M (2021). Comparison of serum VEGF, IGF-1, and HIF-1α levels in children with autism spectrum disorder and healthy controls. J Autism Dev Disord.

[62] Mills JL, Hediger ML, Molloy CA (2007). Elevated levels of growth-related hormones in autism and autism spectrum disorder. Clin Endocrinol (Oxf).

[63] Li Z, Xiao GY, He CY, Liu X, Fan X, Zhao Y, Wang NR (2022). Serum levels of insulin-like growth factor-1 and insulin-like growth factor binding protein-3 in children with autism spectrum disorder. Zhongguo Dang Dai Er Ke Za Zhi.

[64] Abedini M, Mashayekhi F, Salehi Z (2022). Analysis of Insulin-like growth factor-1 serum levels and promoter (rs12579108) polymorphism in the children with autism spectrum disorders. J Clin Neurosci.

[65] Yokoya S, Suwa S, Maesaka H, Tanaka T (1988). Immunoreactive somatomedin C/insulin-like growth factor I in urine from normal subjects, pituitary dwarfs, and acromegalics. Pediatr Res.

[66] Wu WL, Hsiao EY, Yan Z, Mazmanian SK, Patterson PH (2017). The placental interleukin-6 signaling controls fetal brain development and behavior. Brain Behav Immun.

[67] Al-Shanti N, Stewart CE (2012). Inhibitory effects of IL-6 on IGF-1 activity in skeletal myoblasts could be mediated by the activation of SOCS-3. J Cell Biochem.

[68] Umran RM, Al-Tahir M, Jagdish D, Chouthai N (2016). Insulin-like growth factor-1 levels in term newborns with hypoxic-ischemic encephalopathy. Am J Perinatol.

[69] Liu G, Wu HW, Li ZG (2018). Study on the correlation of changes of IGF-1, GH, and NGB levels and NBNA score in neonates with hypoxic ischemic encephalopathy. Eur Rev Med Pharmacol Sci.

[70] Driscoll DJ', Felice VD, Kenny LC, Boylan GB, O'Keeffe GW (2018). Mild prenatal hypoxia-ischemia leads to social deficits and central and peripheral inflammation in exposed offspring. Brain Behav Immun.

[71] Cheroni C, Caporale N, Testa G (2020). Autism spectrum disorder at the crossroad between genes and environment: contributions, convergences, and interactions in ASD developmental pathophysiology. Mol Autism.

[72] Linker SB, Mendes AP, Marchetto MC (2020). IGF-1 treatment causes unique transcriptional response in neurons from individuals with idiopathic autism. Mol Autism.

[73] Pinto D, Pagnamenta AT, Klei L (2010). Functional impact of global rare copy number variation in autism spectrum disorders. Nature.

[74] Zaslavsky K, Zhang WB, McCready FP (2019). SHANK2 mutations associated with autism spectrum disorder cause hyperconnectivity of human neurons. Nat Neurosci.

[75] Chen ST, Lai WJ, Zhang WJ, Chen QP, Zhou LB, So KF, Shi LL (2020). Insulin-like growth factor 1 partially rescues early developmental defects caused by SHANK2 knockdown in human neurons. Neural Regen Res.

[76] Stessman HA, Xiong B, Coe BP (2017). Targeted sequencing identifies 91 neurodevelopmental-disorder risk genes with autism and developmental-disability biases. Nat Genet.

[77] Levy JA, LaFlamme CW, Tsaprailis G, Crynen G, Page DT (2021). DYRK1A mutations cause undergrowth of cortical pyramidal neurons via dysregulated growth factor signaling. Biol Psychiatry.

[78] Fyke W, Velinov M (2021). FMR1 and autism, an intriguing connection revisited. Genes (Basel).

[79] Wang T, Guo H, Xiong B (2016). De novo genic mutations among a Chinese autism spectrum disorder cohort. Nat Commun.

[80] Wen Z, Cheng TL, Li GZ (2017). Identification of autism-related MECP2 mutations by whole-exome sequencing and functional validation. Mol Autism.

[81] Li Y, Wang H, Muffat J (2013). Global transcriptional and translational repression in human-embryonic-stem-cell-derived Rett syndrome neurons. Cell Stem Cell.

[82] Tropea D, Giacometti E, Wilson NR (2009). Partial reversal of Rett syndrome-like symptoms in MeCP2 mutant mice. Proc Natl Acad Sci U S A.

[83] Chen G, Yu B, Tan S (2022). GIGYF1 disruption associates with autism and impaired IGF-1R signaling. J Clin Invest.

[84] Badaloni A, Casoni F, Croci L (2019). Dynamic expression and new functions of early B cell factor 2 in cerebellar development. Cerebellum.

[85] Croci L, Chung SH, Masserdotti G (2006). A key role for the HLH transcription factor EBF2COE2,O/E-3 in Purkinje neuron migration and cerebellar cortical topography. Development.

[86] Croci L, Barili V, Chia D (2011). Local insulin-like growth factor I expression is essential for Purkinje neuron survival at birth. Cell Death Differ.

[87] Zhao B, Zhao CZ, Zhang XY (2012). The new P2Y-like receptor G protein-coupled receptor 17 mediates acute neuronal injury and late microgliosis after focal cerebral ischemia in rats. Neuroscience.

[88] Lin KN, Zhang K, Zhao W, Huang SY, Li H (2022). Insulin-like growth factor 1 promotes cell proliferation by downregulation of G-protein-coupled receptor 17 expression via PI3K/Akt/FoxO1 signaling in SK-N-SH cells. Int J Mol Sci.

[89] Zhong J, Zhao L, Du Y, Wei G, Yao WG, Lee WH (2009). Delayed IGF-1 treatment reduced long-term hypoxia-ischemia-induced brain damage and improved behavior recovery of immature rats. Neurol Res.

[90] Antoine MW, Langberg T, Schnepel P, Feldman DE (2019). Increased excitation-inhibition ratio stabilizes synapse and circuit excitability in four autism mouse models. Neuron.

[91] Unichenko P, Yang JW, Kirischuk S (2018). Autism related neuroligin-4 knockout impairs intracortical processing but not sensory inputs in mouse barrel cortex. Cereb Cortex.

[92] Harrington AJ, Raissi A, Rajkovich K (2016). MEF2C regulates cortical inhibitory and excitatory synapses and behaviors relevant to neurodevelopmental disorders. Elife.

[93] Marchetto MC, Belinson H, Tian Y (2017). Altered proliferation and networks in neural cells derived from idiopathic autistic individuals. Mol Psychiatry.

[94] Bozdagi O, Tavassoli T, Buxbaum JD (2013). Insulin-like growth factor-1 rescues synaptic and motor deficits in a mouse model of autism and developmental delay. Mol Autism.

[95] Matta SM, Hill-Yardin EL, Crack PJ (2019). The influence of neuroinflammation in autism spectrum disorder. Brain Behav Immun.

[96] Zhang P, Omanska A, Ander BP, Gandal MJ, Stamova B, Schumann CM (2023). Neuron-specific transcriptomic signatures indicate neuroinflammation and altered neuronal activity in ASD temporal cortex. Proc Natl Acad Sci U S A.

[97] Fischer I, Shohat S, Levy G, Bar E, Trangle SS, Efrati S, Barak B (2022). Hyperbaric oxygen therapy alleviates social behavior dysfunction and neuroinflammation in a mouse model for autism spectrum disorders. Int J Mol Sci.

[98] Bhalla S, Mehan S (2022). 4-hydroxyisoleucine mediated IGF-1/GLP-1 signalling activation prevents propionic acid-induced autism-like behavioural phenotypes and neurochemical defects in experimental rats. Neuropeptides.

[99] Sha Y, Chen L, Xu C, Zhang B, Hong H, Wang C (2023). Hyperbaric oxygen therapy alleviates social behavior dysfunction and neuroinflammation in a mouse model for autism spectrum disorders. Curr Protein Pept Sci.

[100] Pinto-Benito D, Paradela-Leal C, Ganchala D, de Castro-Molina P, Arevalo MA (2022). IGF-1 regulates astrocytic phagocytosis and inflammation through the p110α isoform of PI3K in a sex-specific manner. Glia.

[101] Wan Y, Gao W, Zhou K, Liu X, Jiang W, Xue R, Wu W (2022). Role of IGF-1 in neuroinflammation and cognition deficits induced by sleep deprivation. Neurosci Lett.

[102] Pangrazzi L, Balasco L, Bozzi Y (2020). Oxidative stress and immune system dysfunction in autism spectrum disorders. Int J Mol Sci.

[103] Usui N, Kobayashi H, Shimada S (2023). Neuroinflammation and oxidative stress in the pathogenesis of autism spectrum disorder. Int J Mol Sci.

[104] Bjørklund G, Meguid NA, El-Bana MA (2020). Oxidative stress in autism spectrum disorder. Mol Neurobiol.

[105] Manivasagam T, Arunadevi S, Essa MM, SaravanaBabu C, Borah A, Thenmozhi AJ, Qoronfleh MW (2020). Role of oxidative stress and antioxidants in autism. Adv Neurobiol.

[106] Rose S, Bennuri SC, Wynne R, Melnyk S, James SJ, Frye RE (2017). Mitochondrial and redox abnormalities in autism lymphoblastoid cells: a sibling control study. FASEB J.

[107] Takahashi S, Hisatsune A, Kurauchi Y, Seki T, Katsuki H (2016). Insulin-like growth factor 1 specifically up-regulates expression of modifier subunit of glutamate-cysteine ligase and enhances glutathione synthesis in SH-SY5Y cells. Eur J Pharmacol.

[108] Hayes CA, Ashmore BG, Vijayasankar A, Marshall JP, Ashpole NM (2021). Insulin-like growth factor-1 differentially modulates glutamate-induced toxicity and stress in cells of the neurogliovascular unit. Front Aging Neurosci.

[109] Fintini D, Brufani C, Cappa M (2009). Profile of mecasermin for the long-term treatment of growth failure in children and adolescents with severe primary IGF-1 deficiency. Ther Clin Risk Manag.

[110] Costales J, Kolevzon A (2016). The therapeutic potential of insulin-like growth factor-1 in central nervous system disorders. Neurosci Biobehav Rev.

[111] Vahdatpour C, Dyer AH, Tropea D (2016). Insulin-like growth factor 1 and related compounds in the treatment of childhood-onset neurodevelopmental disorders. Front Neurosci.

[112] Neul JL, Percy AK, Benke TA (2023). Trofinetide for the treatment of Rett syndrome: a randomized phase 3 study. Nat Med.

[113] Khwaja OS, Ho E, Barnes KV (2014). Safety, pharmacokinetics, and preliminary assessment of efficacy of mecasermin (recombinant human IGF-1) for the treatment of Rett syndrome. Proc Natl Acad Sci U S A.

[114] Glaze DG, Neul JL, Kaufmann WE (2019). Double-blind, randomized, placebo-controlled study of trofinetide in pediatric Rett syndrome. Neurology.

[115] Frank Y (2021). The neurological manifestations of Phelan-McDermid syndrome. Pediatr Neurol.

[116] Kolevzon A, Bush L, Wang AT (2014). A pilot controlled trial of insulin-like growth factor-1 in children with Phelan-McDermid syndrome. Mol Autism.

[117] Yu TW, Berry-Kravis E (2014). Autism and fragile X syndrome. Semin Neurol.

[118] Berry-Kravis E, Horrigan JP, Tartaglia N (2020). A double-blind, randomized, placebo-controlled clinical study of trofinetide in the treatment of fragile X syndrome. Pediatr Neurol.

